# Mechanical, water contact angle and fiber thickness data for Insulin-like growth gactor-1 (IGF-1) incorporated in electrospun random DegraPol^Ⓡ^ fibers and IGF-1 impact on tenocyte aspect ratio and gene expression data

**DOI:** 10.1016/j.dib.2024.111139

**Published:** 2024-11-17

**Authors:** Julia Rieber, Gabriella Meier-Bürgisser, Iris Miescher, Franz E. Weber, Petra Wolint, Yao Yang, Esteban Ongini, Athanasios Milionis, Jess G. Snedeker, Maurizio Calcagni, Johanna Buschmann

**Affiliations:** a Division of Plastic Surgery and Hand Surgery, University Hospital Zurich, Sternwartstrasse 14, 8091 Zurich, Switzerland; b Oral Biotechnology & Bioengineering, Center for Dental Medicine, Cranio-Maxillofacial and Oral Surgery, University of Zurich, Zurich 8032, Switzerland; c Department of Health Sciences & Technology & Department of Materials, Schmelzbergstrasse 9, LFO, 8092 Zürich, Switzerland; d University Clinic Balgrist, Orthopaedic Biomechanics, Forchstrasse 340, 8008 Zurich, Switzerland; e Laboratory of Thermodynamics in Emerging Technologies, Department of Mechanical and Process Engineer-ing, ETH Zürich, 8092 Zürich, Switzerland

**Keywords:** Ultimate tensile stress, Young's modulus, Ultimate fracture strain, Hydrophobicity, Rabbit Achilles tenocytes, Collagen type I, ki67, Tenomodulin

## Abstract

The first set of data refers to Insulin-like Growth Factor-1 (IGF-1) protein incorporation via emulsion electrospinning into a DegraPol^Ⓡ^ random fiber mesh and its characterization. Specifically, the fiber thickness was assessed and compared to pure DegraPol^Ⓡ^ fibers without IGF-1 (control). Furthermore, the mechanical properties of these meshes were assessed and data on ultimate tensile stress, Young's modulus and ultimate fracture strain are presented for ring specimen and rectangular pieces taken from electrospun tubes in the transverse direction as well as rectangular pieces taken in the axial direction of the electrospun tube. Moreover, the static and the dynamic water contact angles were determined.

The second set of data represents morphological aspects, such as the cytoskeletal aspect ratio (i.e. length of the cell divided by its width) for rabbit Achilles tenocytes stimulated *in vitro* with 1, 10, and 100 ng/mL IGF-1 supplementation compared to the corresponding cell culture without IGF-1 (control). Furthermore, qPCR was performed and collagen I, ki67 and tenomodulin gene expression data are presented for rabbit Achilles tenocytes in vitro with 0.1, 1 and 10 ng/mL IGF-1 supplementation, respectively, as well as with a supplementation of released IGF-1 from the DegraPol mesh (concentration was 1 ng/mL).

Specifications Table

**Table:** 

Subject	Material sciences; genetics; cell biology
Specific subject area	Emulsion electrospun DegraPol^Ⓡ^ fiber meshes with incorporated IGF-1 (water-in-oil emulsion) were characterized. Using scanning electron microscopy, fiber thickness was assessed. With a tensile testing machine, ultimate tensile stress, Young's modulus and ultimate fracture strain were measured. Furthermore, data for water contact angles (static and dynamic) are presented. Cell culture data were determined for monolayer tenocyte cultures. Tenocytes were harvested from freshly isolated New Zealand White Rabbit Achilles tendons of four rabbits. These monolayer tenocyte cultures were supplemented with the growth factor IGF-1 (0.1, 1 and 10 ng/mL IGF-1 and 1 ng/mL IGF-1 that was released from an emulsion electrospun mesh with incorporated IGF-1 to test if the released IGF-1 was still bioactive), and gene expression for *collagen I, ki67* and *tenomodulin* was assessed after 3 days (control no IGF-1). The aspect ratio of the cells was assessed for 1, 10, and 100 ng/mL IGF-1 supplementation and compared to the control without IGF-1.
Data format	The data consist of raw data and of analysed data (mean and standard deviation of the mean).
Type of data	Excel files of the type *.xlsx* files (data sets with group labels and numbers)
Data collection	**Scanning electron microscopy (SEM)**: A SEM was used (Zeiss Gemini SEM 450) and images of the outer and the inner surfaces of electrospun meshes were used to assess the fiber thickness. **Mechanics**: For the mechanical assessment, a uniaxial tensile testing machine (Zwick Z010 with a 20 N load cell) was used. **Water contact angles (WCA)**: Static and dynamic WCA on the material surfaces were measured by a video-based optical contact angle measuring instrument OCA 35 Dataphysics, Germany. **qPCR**: Rabbit Achilles tenocytes were cultured as monolayers and RNA was extracted by an **RNeasy Plus Mini Kit** from Qiagen, Hilden, Germany; followed by reverse transcription with the **iScript Advanced cDNA Synthesis Kit** (Bio-Rad, Cressier, Switzerland). The quantitative real-time PCR reactions were performed with **CFX Connect Real-Time PCR** Detection System (Bio-Rad, Cressier, Switzerland) and **SsoAdvanced SYBR Green Supermix** (Bio-Rad, Cressier, Switzerland). **Aspect ratio**: A microscope was utilized to analyze the cell length and width (Leica Microscope 6000 B, Leica Camera AG, Germany).
Data source location	Gene expression, aspect ratio, fiber thickness data were collected in Zurich, Switzerland, at the University Hospital Zurich. Water contact angles were determined in Zurich, Switzerland, at the ETH. Mechanical data were collected in Zurich, Switzerland, at the Balgrist Campus (University Hospital Balgrist).
Data accessibility	**Repository name**: Mendeley Data **Data identification number**: 10.17632/ssvg5shrw6.1 and 10.17632/79vtb857mk.1 **Direct URL** to data: Fiber thickness, water contact angle and mechanical properties of electrospun DegraPol^Ⓡ^ and emulsion electrospun DegraPol^Ⓡ^/Insulin-like growth factor-1 (IGF-1) fiber meshes - Mendeley Data Impact of Insulin-like growth factor-1 (IGF-1) on the aspect ratio and gene expression of rabbit Achilles tenocytes - Mendeley Data
Related research article	Bioactive and Elastic Emulsion Electrospun DegraPol^Ⓡ^ Tubes Delivering IGF-1 for Tendon Rupture Repair - PubMed (nih.gov) [1]

## 1 Value of the Data

• Fiber thickness data may be reused for comparison with other polymer fiber meshes fabricated by electrospinning; particularly of interest for material scientists will be the influence of emulsion electrospinning versus single electrospinning (pure polymer fiber).
• Mechanical data of electrospun tubes in axial and transverse directions may be reused to study and simulate anisotropy of electrospun fibers in a cylindrical architecture.
• Mechanical data of electrospun tubes made from DegraPol^Ⓡ^ polymer may be reused to compare them to other polymers.
• Water contact angle data can be reused to compare to other polymers than DegraPol^Ⓡ^ and may be used to collect many different polymers’ water contact angles as lists for hydrophobicity characteristics in reference books.
• These data can be reused, if other cell types than tenocytes are stimulated with IGF-1 *in vitro* – for a direct comparison of their collagen I, ki67 and tenomodulin gene expression and morphological features.
• These data can be reused, if tenocytes from other species than rabbits are stimulated with IGF-1 *in vitro* to be compared to our data determined for rabbit tenocytes.
• These data can be reused for experimental comparison of rabbit Achilles tenocyte stimulation with other growth factors than IGF-1, if collagen I, ki67 and tenomodulin gene expression and/or morphology of the cells is investigated.
• The gene expression data and the morphology data can be reused for experimental comparison of rabbit Achilles tenocyte treatment with drugs aimed at tendon healing, such as aspirin [2], curcumin [3], or others to be compared to an IGF-1 supplementation.
• Our data may help researchers in elucidating signaling mechanisms relating to cell growth, motility, or other pathways, where IGF-1 plays a regulatory role as a growth factor.

## 2 Background

Besides adhesion formation to the surrounding tissue, the major problem in surgical tendon repair is re-rupture caused by fibrovascular scar formation, ending up in inferior mechanical properties after healing. To counteract these problems, the design of novel implant materials has envisioned incorporated growth factors to be slowly released to the wound site and to support tendon healing with the aim to provide stronger tendons at earlier time points post operation. Among them, IGF-1 is of particular interest because of its reported beneficial effects, such as increased cell proliferation, enhanced DNA expression and pronounced extracellular matrix formation [1,4]. In addition, electrospinning techniques, with multiple options, such as single electrospinning [5], coaxial electrospinning [6] or even triaxial, and side-by-side processes [7] have gained much attention. Therefore, IGF-1 was incorporated into electrospun DegraPol^Ⓡ^ fiber meshes from a water-in-oil emulsion, because DegraPol^Ⓡ^ as a biocompatible and pH neutrally biodegradable co-block polymer has been shown promising when fabricated as electrospun tube to be applied over conventionally sutured rabbit Achilles tendons [8,9]. Such DegraPol^Ⓡ^ tubes with incorporated IGF-1 were characterized with respect to fiber thickness, mechanics and water contact angles.

## 3 Data Description

The data are stored as Microsoft Excel (Microsoft Corporation, Redmond, WA, USA) files (.xlsx files) in the Mendeley Data repository service Fiber thickness, water contact angle and mechanical properties of electrospun DegraPol^Ⓡ^ and emulsion electrospun DegraPol^Ⓡ^/Insulin-like growth factor-1 (IGF-1) fiber meshes - Mendeley Data and Impact of Insulin-like growth factor-1 (IGF-1) on the aspect ratio and gene expression of rabbit Achilles tenocytes - Mendeley Data.

The first repository Fiber thickness, water contact angle and mechanical properties of electrospun DegraPol^Ⓡ^ and emulsion electrospun DegraPol^Ⓡ^/Insulin-like growth factor-1 (IGF-1) fiber meshes - Mendeley Data includes three excel files, named Fiber Thickness.xlsx with four sheets; Mechanics_IGF-1 mesh.xlsx with one sheet and Water contact angles.xlsx with two sheets, respectively.

1. Characterization of DegraPol^Ⓡ^ tubes with incorporated IGF-1

**File 1** Fiber Thickness.xlsx

This excel file is open access published in Mendeley Data Fiber thickness, water contact angle and mechanical properties of electrospun DegraPol^Ⓡ^ and emulsion electrospun DegraPol^Ⓡ^/Insulin-like growth factor-1 (IGF-1) fiber meshes - Mendeley Data and contains four sheets, called *Pure DP, DP-IGF-1, DP-PDGF-BB* and *DP-both*, respectively. These four sheets are arranged the same, so we will describe the content of the columns only once. In column A, there is the kind of material in the first field and below this first field there is the specification if the outer or the inner surface of the electrospun tube was analyzed, so the reader knows if SEM images of the outer surface which is obtained at the end of electrospinning was used or otherwise SEM images of the inner surface which is the surface that faced the flat target/collector. In column C, the file name of the SEM image is given, which was the basis to determine the fiber thickness. In column D, the reference distance measured in the image and in column E the true distance is given in µm. In column F, the number of each single fiber analyzed in a particular SEM image is given. In column G, the measured thickness is given and in column H the calculated absolute fiber thickness is shown. In column I the mean and in column J the standard deviation of the mean are given for the fiber thickness assessed in a specific image (see name of image in column C). In column K and in column L the mean and standard deviation for a specific condition, such as for outer or inner surface, is calculated.

**File 2** Mechanics_IGF-1 mesh.xlsx

**Fig. 1:**
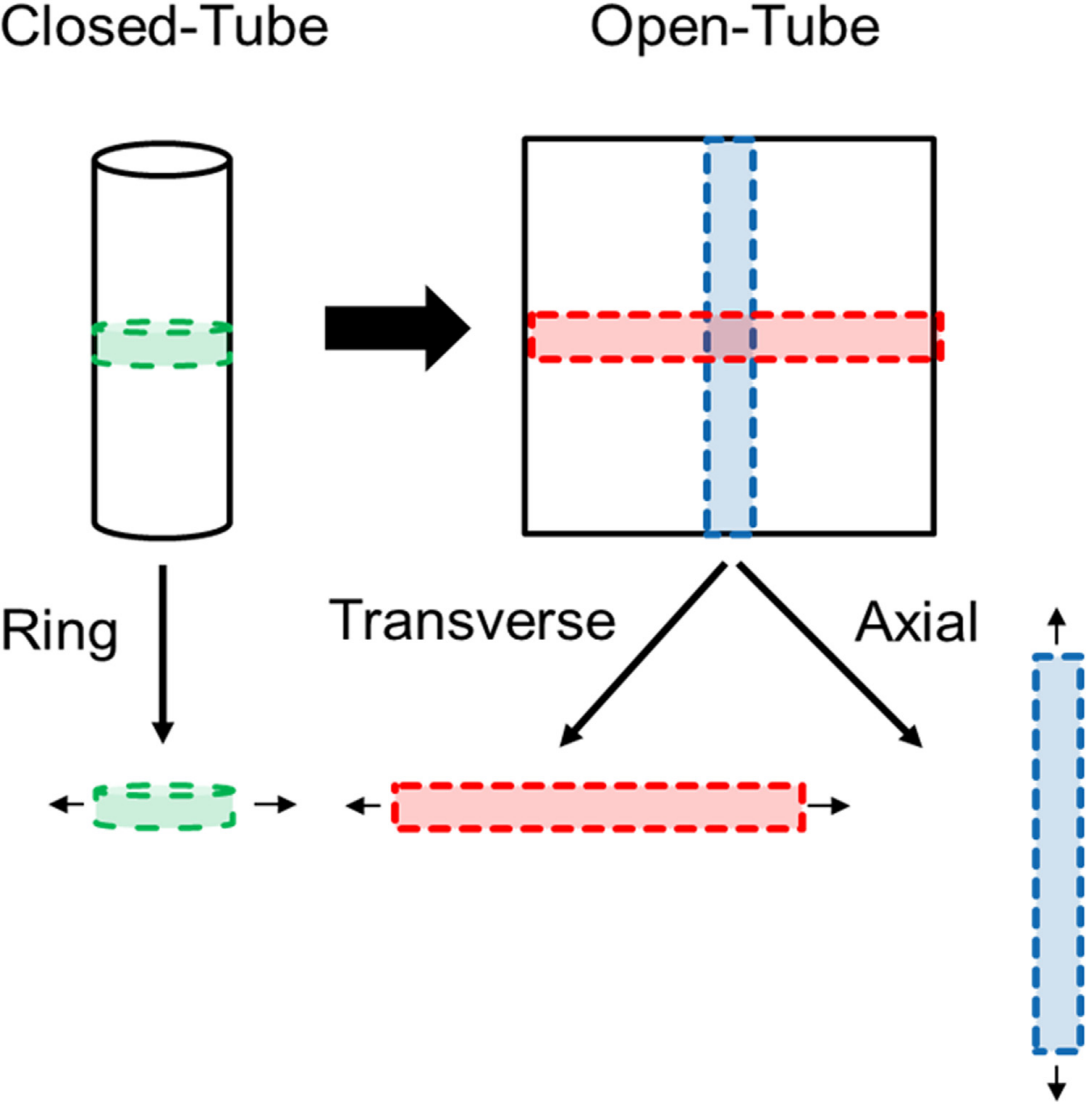
The mechanics of the tubular electrospun mesh were tested in transverse direction as ring (green), in transverse direction as rectangular piece (red) and in axial direction as rectangular piece (blue).

This excel file is open access published in Mendeley Data Fiber thickness, water contact angle and mechanical properties of electrospun DegraPol^Ⓡ^ and emulsion electrospun DegraPol^Ⓡ^/Insulin-like growth factor-1 (IGF-1) fiber meshes - Mendeley Data and contains one sheet called *Mechanical parameters*. In the first row, the direction for tensile testing is given, with ring = ring specimen tested in transverse direction; transverse = rectangular piece cut out of the tube in transverse direction tested in transverse direction; or axial = rectangular piece cut out of the tube in axial direction tested in axial direction (Fig. 1). In the second row, the material is given; either DP = DegraPol^Ⓡ^ pure or DP-IGF-1 = IGF-1 incorporated in DegraPol^Ⓡ^. In column A below the second row, the tested parameter is given and in square brackets the entity of the respective parameter is shown.

**File 3** Water contact angles.xlsx

This excel file is open access published in Mendeley Data Fiber thickness, water contact angle and mechanical properties of electrospun DegraPol^Ⓡ^ and emulsion electrospun DegraPol^Ⓡ^/Insulin-like growth factor-1 (IGF-1) fiber meshes - Mendeley Data and contains two sheets called *Static WCA* and *Dynamic WCA*, respectively. In the first sheet called *Static WCA*, in column A, the kind of electrospun fiber mesh is denoted; either it is pure DegraPol^Ⓡ^ or it is DegraPol^Ⓡ^ with incorporated IGF-1. In column B, the right static WCA angle is given for tube 1. In column C, the left static WCA is given for tube 1. In column D, the mean of the static WCA on the right and on the left of the water droplet is calculated. The same is given in columns G-I for tube 2; and in columns l-N for tube 3, respectively. In the second sheet called *Dynamic WCA*, in column A, the kind of tube is specified; either it is pure DegraPol^Ⓡ^ or it is DegraPol^Ⓡ^ with incorporated IGF-1 and then the right WCA of tube 1 is shown, either for the advancing dynamic WCA or for the receding dynamic WCA, respectively. In column B, the left dynamic WCA is given and in column C, mean of right and left dynamic WCAs is calculated. Furthermore, in column D, the standard deviation of the three calculated means of right and left dynamic WCAs is calculated and finally In column E the hysteresis = advancing minus receding WCA is given. For columns H-L, the same is given for tube 2; and for columns O-S the same is shown for the third tube (tube 3). In the columns U-Z, the mean, standard deviation of the three tubes are depicted.

2. Gene expression and aspect ratio under IGF-1 supplementation to tenocytes

The second repository called Impact of Insulin-like growth factor-1 (IGF-1) on the aspect ratio and gene expression of rabbit Achilles tenocytes - Mendeley Data contains **two** Excel files named *Aspect Ratio_IGF-1 supplementation.xlsx and qPCR_IGF-1 supplementation.xlsx*.

**File** Aspect Ratio_IGF-1 supplementation.xlsx with one sheet called *Aspect Ratio*

In the sheet *Aspect Ratio*, in column A the cells are numbered for the first condition (0 ng/mL IGF-1). In column B, the width of the cells are shown in µm, while in column C the length of the cells are given in µm. In column D, the aspect ratio with width divided by length and in column E the aspect ratio with length divided by width is presented. The same order of data for the second condition (1 ng/ml IGF-1) is found in columns G-K; the same order for the third condition (10 ng/ml IGF 1) is found in columns M-Q; and the same order for the fourth condition (100 ng/ml IGF-1) is found in columns S-W.

**File 2** qPCR_IGF-1 supplementation.xlsx with one sheet named *2^-ddCT Mean*

This excel file is open access published in Mendeley Data Impact of Insulin-like growth factor-1 (IGF-1) on the aspect ratio and gene expression of rabbit Achilles tenocytes - Mendeley Data and contains one sheet called *2^-ddCT Mean.* In the first row, the IGF-1 concentration and the mode are given; either freshly supplied or from a harvested in the supernatant from a release experiment, where IGF-1 was released from an electrospun tube with incorporated IGF-1.In column A, the target gene and the rabbit donor number are given. In column B, the 2^-ddCT Mean values are given for the 0 ng/mL IGF-1 supplementation experiment. In column C, the 2^-ddCT Mean values are given for the 0.1 ng/mL IGF-1 supplementation experiment, while these values are shown in column D for the 1 ng/mL IGF-1 supplementation experiment and in column E for the 10 ng/mL IGF-1 supplementation experiment. Column F represents the 2^-ddCT Mean values for 1 ng/mL IGF-1 release experiment; in other words the IGF-1 was first harvested from a release experiment where the IGF-1 incorporated electrospun DegraPol fiber mesh was in PBS and the released IGF-1 was collected and supplemented to the tenocytes in a concentration of 1 ng/mL IGF-1.

## 4 Experimental Design, Materials and Methods

### 4.1 Fabrication of electrospun meshes

DegraPol^Ⓡ^ (DP) was synthesized with the following fractions of components by dissolution in 1,4-dioxane and before drying it to a water content lower than 20 ppm: 25 wt% of poly (3-(R-hydroxybutyrate)-co-(ε-caprolactone)-diol (Mn = 2824 g mol^−1^) and 75 wt% of poly(ε-caprolactone)-diol-co-glycolide (15 mol% glycolide, 85 mol% ε-caprolactone) (Mn= 1000 g mol^−1^). After cooling the solution, an amount of 2,2,4-trimethylhexane-diisocyanate (TMDI) was added that corresponded to the stochiometry. Dibutyltin dilaurate (20 ppm) was added 3 times the next day within one day to reach a final molecular weight of 100–110 kDa. Cooled hexane was utilized to precipitate the polymer isomers. Finally, they were purified with chloroform and silicagel 60 column (Fluka), with subsequent precipitation in cooled ethanol.

One to 3 days before electrospinning, all solutions were prepared. First, a polyethylene glycol (PEG) (35 kDa, Sigma-Aldrich, Germany #81310) solution was prepared for each scaffold, by mingling 3.5 g of chloroform (Sigma-Aldrich, Germany, #132950) and 1.5 g of PEG. Then, a DP solution was made with 0.6 g of DP powder, 0.88 g of 1,1,1,3,3,3-Hexafluoro-2-propanol (HFP, Sigma-Aldrich, Germany, #105228) and 3.52 g chloroform into a dark glass with screw cap. The IGF-1 solution required recombinant human IGF-1 (PeproTech, USA, #100-11-100UG), and a total amount of 4 µg IGF-1 dissolved in 400 µL phosphate buffered solution (PBS, BioConcept, Switzerland, #3-05F39-I) including also rabbit serum albumin (RSA; 10 µg/mL IGF-1 and 0.25 % RSA in PBS) was added drop by drop to the DP solution to produce a water-in-oil emulsion, with subsequent stirring for five minutes on a magnetic stirrer (500 rpm). The solution was heavily mixed on the vortex and then additionally emulsified in an ultrasonic bath for 15 min. After filling the emulsion in a 5 mL glass syringe (Huberlab,# 3.7102.33), it was electrospun right away. Homogenous distribution of water droplets containing IGF-1 within the DP fibers can be assumed because this protocol to generate a water-in-oil emulsion with IGF-1 was the same as used in another study for the incorporation of the growth factor PDGF-BB [10].

We used an in-house assembly for electrospinning, equipped with a DC high voltage supply (Glassman High Voltage Inc., High Bridge, NJ, US), a needle holder, transporter and a syringe pump (SP210cZ, WPI, Germany). A needle (1 mm inner diameter and 0.3 mm wall thickness) made of stainless steel (Angst & Pfister AG, Zürich, Switzerland) was included in the spinning head having a blunt end. A metal rod served as collector, which was 55 cm long. It was mounted on the rotary motor (the Euro Star B rotary motor, IKA Labortechnik). The flow rate was 1 mL/h, the working distance was 19.5 cm between the spinning needle and the metal rod, and the voltage was 12.5 kV voltage. The collector rotated at a speed of 500 rpm. The conditions in the room were kept at 22–23 °C and 25–35 % relative moisture. The needle was moving constantly sideward to the left and to the right in a total range of 20 cm in order to transport the DP solution evenly over the target. A first layer of PEG was deposited on the metal rod to facilitate the detachment of the DP tube with 50 % ethanol. Subsequently, the DP or DP with IGF-1 layers were electrospun on top of this PEG layer. All tubes were stored in a desiccator. Dry storage is highly important because hydrolysis may occur.

### 4.2 Scanning electron microscopy (SEM)

Samples were prepared as follows with the aim of visualizing the inner and outer surfaces of the electrospun mesh. First, the samples were fixed on a SEM carrier with conductive double-sided adhesive. The surface coating and imaging was performed with equipment provided by the Center for Microscopy and Image Analysis at University in Zurich (CH). The probes were coated with a 10 nm film of platinum utilizing a sputter coater (Safematic CCU-010). The machine used to take images was a SEM (Zeiss Gemini SEM 450) and the voltage was 5 kV. Visualization was performed at a magnification of 507 x and at a brightness of 49 %. The mode of the detector was set to “secondary electrons”. Fiber thickness was measured by ImageJ (1.53e / Java 1.8.0_172 (64-bit)) using the scale bar from the microscope image as a reference length. For each SEM picture, a diagonal line was drawn and all the fibers that were crossed by this line were measured with respect to their thickness.

### 4.3 *Mechanics*

Utilizing a uniaxial tensile testing machine (Zwick Z010 with a 20 N load cell), stress/strain curves were gauged. The axial and transverse direction were measured, using rectangular specimen cut from 6 mm tubes with an area of 36 mm^2^ (2 mm x 18 mm). At the beginning, the gauge length between clamps was 10 mm. For the measurements of the rings, the tubes were cut into 2 mm pieces and clamped to an initial gauge length of 8 mm. As the thickness of these pieces was different, only the material properties were assessed and compared. At a strain rate of 10 mm/min, every sample was stretched until failure. The strain at break [%], ultimate tensile stress [MPa] and Young's modulus [MPa] were determined for every condition (*n* = 6).

### 4.4 Water contact angles

The measurement of water contact angle (WCA) for pure DP and DP emulsion electrospun meshes with IGF-1 was taken. Static and dynamic contact angles were measured on the corresponding surfaces by a video-based optic contact angle determining instrument: OCA 35 Dataphysics, Germany. A drop with a volume of 5 µL was used for each measurement, according to WCA measurements reported earlier [11]. The samples were placed under the syringe (1 mL) filled with water and a drop was gently placed on the surface. The left and right angles between the water drop and the surface were determined. The mean values of these angles was computed and denoted as WCA, whereby three values were averaged per condition (3 technical replicates).

The advancing WCA was similarly assessed. One initial static drop (5 µL) was filled continuously with milli-Q water and when the baseline (the area where the drop was in contact with the surface of the material) was getting larger, the advancing angle was taken and computed. The receding angle was gauged accordingly, i.e. when the baseline was getting smaller (suddenly) during the slow removal of water. The contact angle hysteresis was then computed by the advancing angle minus the receding WCA [12]. For that purpose, a video of the process was taken and the left and right angles were quantified by ImageJ software (1.53e / Java 1.8.0_172 (64-bit)) and the mean of them was taken. Three repetitions were made for each condition (*n* = 3).

### 4.5 In vitro rabbit Achilles tenocyte culture

Rabbit tenocytes were extracted from Achilles tendons of female New Zealand White rabbits that had an age between 12 and 16 weeks utilizing a method of cell migration (Veterinary license of Canton Zurich, reference number 255/15). To do so, tendons were extracted from the rabbits and they were first washed with Hank's Balanced Salt Solution (1x HBSS with Ca^2+^ and Mg^2+^, Thermo Fisher Scientific, Rockford, IL, USA) with 200 µg/mL gentamycin (Biowest, Nuaillé, France) as well as with the antibiotic amphotericin B (Biowest, Nuaillé, France) applied in a concentration of 2.5 µg/mL. Adjacent tissue was removed, and the middle area of the tendons was cut into mini pieces (< 2 mm) and washed three times with 1x HBSS buffer. Afterwards, several pieces of tissue were transferred into a tissue culture plate (PrimariaTM, Corning, New York, NY, USA) and a drop of cell culture medium (Ham's F12 (Biowest, Nuaillé, France), 10 % FBS (Biowest, Nuaillé, France), 200 µg/mL gentamycin, and 2.5 µg/mL amphotericin B) was added onto each tissue piece. Tissues could attach on the cell culture plates for 2 h at 37 °C and 5 % CO_2,_ prior to addition of 10 mL of cell culture medium to each tissue culture plate.

The tissue culture plates with the tendon pieces were not moved within the first five days, to minimize and avoid tendon tissue detachment from the bottom of the plates and to allow cells to start migrating out undisturbedly from these tissues. The first medium change was undertaken after five days. Later than that, the culture medium was exchanged every third day. After 14 days, the tendon tissue pieces were completely removed from the plates. For a further week, cells could proliferate prior to cryopreservation until further use. For expansion, cryopreserved rabbit tenocytes were thawed, again suspended in culture medium (Ham's F12 with 10 % FBS and 50 µg/mL gentamycin) and cultured at 37 °C and 5 % CO_2_. The culture medium was changed every other day. Only passages 2 and 4 (P2–P4) were used for all in vitro tenocyte culture experiments, because at higher passages there might be a phenotypic drift typical for tendon cells [13]. On day 3, aspect ratio of the cells was assessed by measuring the width and length of the cells in monolayer cell culture images taken by a microscope equipped with a camera (Leica Microscope 6000 B, Leica Camera AG, Germany).

### 4.6 qPCR

After rabbit tenocyte isolation, the cells were put in cultures as described under *In vitro rabbit Achilles tenocyte culture* and they were seeded into 12-well plates (TPP, Trasadingen, Switzerland, growth area per well: 3.60 cm^2^) with a starting cell density of 40,000 cells in each well. The tenocytes were allowed to attach to the well bottom during the night before the medium was changed to medium with IGF-1 supplementation (0.1, 1 and 10 ng/mL were tested as well as released IGF-1 at a concentration of 1 ng/mL) or to control medium without any supplementation (Ham's F12, 10 % FBS, 50 µg/mL gentamycin). The growth factor was added freshly to the medium at every medium exchange. The medium was exchanged every day until day 3 (end of experiment). Rabbit tenocytes from two different animals were used (*n* = 2 donors) and experiments were performed in triplicates for each rabbit donor (*n* = 3).

At day 3, total RNA was extracted utilizing the RNeasy Plus Mini Kit (Qiagen, Hilden, Germany, Switzerland) with RNase-free DNase (Qiagen, Hilden, Germany), using the manufacturer's protocol. Five hundred ng of total RNA was reverse-transcribed into cDNA using a reaction volume of 20 µL and the iScript Advanced cDNA Synthesis Kit (Bio-Rad, Cressier, Switzerland). The PCR reactions were then conducted, taking the corresponding cDNA samples (5 ng cDNA per reaction), and utilizing the CFX Connect Real-Time PCR Detection System (Bio-Rad, Cressier, Switzerland) as well as SsoAdvanced SYBR Green Supermix (Bio-Rad, Cressier, Switzerland). The PCR reactions were done at 95 °C for 3 min, with subsequent 39 cycles of 95 °C for 10 s and 62 °C for 30 s. Two repetitions (n = 2) were performed for each sample. The following primer sequences were used for all measurements: for collagen I (Col1A1) the forward sequence were (5′−3′): CTGGTGAATCTGGAC-GTGAG and the reverse sequence TGTCTCACCCTT-GTCACCAC; for tenomodulin forward sequences were (5′−3′): GCAGTTTCCGAG-TTACAAGAC and reverse CGACGGCAG-TAAATACAACAG; for ki67 forward sequences were (5′−3′): CACATCCAGCAG-TGAAACGG and reverse GTGTTAGCAGTAC—CTGAAGTC. As reference gene, 18S was used with forward were (5′−3′): GGAACTGAGGCCATGATTAAG and reverse CGGAACTACGACGG-TATCTG primer sequences. All used primers were purchased from Microsynth, Balgach, Switzerland. Relative target gene expression analysis was performed with the comparative 2^-ΔΔCT^ method [14]. The results are presented as fold change normalized to the control, compared to samples cultivated without IGF-1: The condition without IGF-1 was set to 1, serving as the control.

## Limitations

Gene expression data were obtained only for target genes encoding collagen I, ki67 and tenomodulin; however, other target genes, such as for collagen III, Mohawk or pro-inflammatory markers like IL-6 may would have been interesting as well.

## Ethics Statement

The rabbit Achilles tenocytes were harvested from rabbits using an animal license approved by the veterinary office of Canton Zurich, Switzerland, having the reference number 255/15.

## CRediT Author Statement

**Julia Rieber:** Conceptualization, Methodology, Data curation, Investigation, Writing – Original draft preparation, Writing – Reviewing and Editing. **Gabriella Meier Bürgisser**: Data curation, Investigation, Writing – Reviewing and Editing. **Iris Miescher**: Data curation, Writing – Reviewing and Editing. **Franz E. Weber**: Supervision, Writing – editing. **Petra Wolint**: Data curation, Writing – Reviewing and Editing. **Yao Yang**: Data curation, Writing – Reviewing and Editing. **Esteban Ongini**: Methodology, Investigation, Data curation, Writing – Reviewing and Editing. **Athanasios Milionis**: Methodology, Investigation, Data curation, Writing – Reviewing and Editing. **Jess G. Snedeker**: Supervision, Writing – Reviewing and Editing. **Maurizio Calcagni**: Supervision, Writing – Reviewing and Editing. **Johanna Buschmann**: Conceptualization, Supervision, Methodology, Writing - Original draft preparation, Writing – Reviewing and Editing, Fund raising.

## Data Availability

Mendeley DataFiber thickness, water contact angle and mechanical properties of electrospun DegraPol^Ⓡ^ and emulsion electrospun DegraPol^Ⓡ^/Insulin-like growth factor-1 (IGF-1) fiber meshes (Original data). Mendeley DataImpact of Insulin-like growth factor-1 (IGF-1) on the aspect ratio and gene expression of rabbit Achilles tenocytes (Original data).

## References

[1] Rieber J., Meier-Bürgisser G., Miescher I., Weber F.E., Wolint P., Yao Y., Ongini E., Milionis A., Snedeker J.G., Calcagni M., Buschmann J. (2023). Bioactive and elastic emulsion electrospun DegraPol tubes Delivering IGF-1 for tendon rupture repair. Int. J. Mol. Sci..

[2] Wang Y., He G., Tang H., Shi Y., Kang X., Lyu J., Zhu M., Zhou M., Yang M., Mu M., Chen W., Zhou B., Zhang J., Tang K. (2019). Aspirin inhibits inflammation and scar formation in the injury tendon healing through regulating JNK/STAT-3 signalling pathway. Cell Prolif..

[3] Chen B., Liang Y., Zhang J., Bai L., Xu M., Han Q., Han X., Xiu J., Li M., Zhou X., Guo B., Yin Z. (2021). Synergistic enhancement of tendon-to-bone healing via anti-inflammatory and pro-differentiation effects caused by sustained release of Mg(2+)/curcumin from injectable self-healing hydrogels. Theranostics.

[4] Miescher I., Rieber J., Calcagni M., Buschmann J. (2023). In Vitro and In Vivo effects of IGF‑1 delivery strategies on tendon healing: a review Int. J. Mol. Sci..

[5] Buschmann J., Calcagni M., Meier Buergisser G., Bonavoglia E., Neuenschwander P., Milleret V., Giovanoli P. (2015). Synthesis, characterization and histomorphometric analysis of cellular response to a new elastic DegraPol (R) polymer for rabbit Achilles tendon rupture repair. J. Tissue Eng. Regen. Med..

[6] Zhou J., Wang W., Yang X., Yu D.-G., Liu P. (2024). Electrospun gelatin/tea polyphenol@pullulan nanofibers for fast-dissolving antibacterial and antioxidant applications. J. Food Sci..

[7] Gong W., Wang M.-L., Liu Y., Yu D.-G., Bligh S.W. (2024). Shell distribution of vitamin K3 within reinforced electrospun nanofibers for improved photo-antibacterial performance. Int. J. Mol. Sci..

[8] Meier-Bürgisser G., Evrova O., Heuberger D.M., Wolint P., Rieber J., Miescher I., Schüpbach R.A., Giovanoli P., Calcagni M., Buschmann J. (2021). Electrospun tube reduces adhesion in rabbit Achilles tendon 12 weeks post-surgery without PAR-2 overexpression. Sci. Rep..

[9] Buschmann J., Meier Buergisser G., Bonavoglia E., Neuenschwander P., Milleret V., Giovanoli P., Calcagni M. (2013). Cellular response of healing tissue to DegraPol tube implantation in rabbit Achilles tendon rupture repair: an in vivo histomorphometric study. J. Tissue Eng. Regen. Med..

[10] Evrova O., Houska J., Welti M., Bonavoglia E., Calcagni M., Giovanoli P., Vogel V., Buschmann J. (2016). Bioactive, elastic, and biodegradable emulsion electrospun DegraPol tube delivering PDGF-BB for tendon rupture repair. Macromol. Biosci..

[11] Hu M., Korschelt K., Viel M., Wiesmann N., Kappl M., Brieger J., Landfester K., Thérien-Aubin H., Tremel W. (2018). Nanozymes in nanofibrous mats with haloperoxidase-like activity to combat biofouling. ACS Appl. Mater. Interfaces.

[12] Eral H.B., ’t Mannetje D.J.C.M., Oh J.M. (2013). Contact angle hysteresis: a review of fundamentals and applications. Colloid Polym. Sci..

[13] Yao L., Bestwick C.S., Bestwick L.A., Maffulli N., Aspden R.M. (2006). Phenotypic drift in human tenocyte culture. Tissue Eng..

[14] Livak K.J., Schmittgen T.D. (2001). Analysis of relative gene expression data using real-time quantitative PCR and the 2(T)(-Delta Delta C) method. Methods.

